# The Effects of Vibratory and Acoustic Stimulations on Postural Control in Healthy People: A Systematic Review

**DOI:** 10.1007/s10439-023-03136-x

**Published:** 2023-01-26

**Authors:** Roberta Minino, Antonella Romano, Emahnuel Troisi Lopez, Marianna Liparoti, Pierpaolo Sorrentino, Antonio Fratini

**Affiliations:** 1 Department of Motor Sciences and Wellness, University of Naples “Parthenope”, Naples, Italy; 2 Department of Social and Developmental Psychology, University of Rome “Sapienza”, Rome, Italy; 3 Institut de Neuroscience des Systemès, Aix-Marseille University, Marseille, France; 4 Department of Mechanical, Biomedical and Design Engineering, Aston University, Aston Triangle, Birmingham, B4 7ET UK

**Keywords:** Sensory stimulations, Acoustic cues, Vibrotactile cues, Balance, Upright stance

## Abstract

Research on human posture and balance control has grown in recent years, leading to continued advances in their understanding. The ability to maintain balance is attributed to the interplay of the visual, vestibular, and somatosensory systems, although an important role is also played by the auditory system. The lack or deficit in any of these systems leads to a reduced stability that may be counterbalanced by the integration of all the remaining sensory information. Auditory and vibratory stimulation have been found to be useful to enhance balance alongside daily activities either in healthy or pathological subjects; nevertheless, while widely investigated, the literature relating to these approaches is still fragmented. This review aims at addressing this by collecting, organising, and discussing all the literature to date on the effects of the various acoustic and vibratory stimulation techniques available on static upright posture in healthy subjects. In addition, this review intends to provide a solid and comprehensive starting point for all the researchers interested in these research areas. A systematic search of the literature was performed and a total of 33 articles (24 on vibratory stimulation and 9 on acoustic stimulation) were included in our analysis. For all articles, several elements were highlighted including: the study sample, the characteristics of the stimulations, the recording instruments, the experimental protocols, and outcomes. Overall, both stimulations analysed were found to have a positive effect on balance but more research is needed to align those alternative approaches to the traditional ones.

## Introduction

From a pure mechanical perspective, human balance can be considered as equivalent to a condition of equilibrium, which is the state of an object when the resultant of the forces acting on it is zero.^56^ Human stance is however intrinsically unstable and constantly influenced by external and internal constraints, which make it necessary for the body to continuously control balance.^45^ This ability depends on sensory and motor processes through which the postural control mechanisms are performed.^21^ In more details: (i) the vestibular system provides the position of the head in space and its linear and angular acceleration; (ii) the visual system is responsible for providing information about the position of the body within the surrounding environment; (iii) the somatosensory system (or proprioception) records the position and movements of each body segment, playing a key role in maintaining balance^12,19^; (iv) finally the auditory system, which, even if rarely considered in balance control, contributes to the perception of the three-dimensionality of the surrounding space and is a supplementary source of information useful for maintaining balance.^66^

It is hypothesised that the integration among the above-mentioned systems^76^ enables balance control in different environmental conditions. However, with ageing the body undergoes physical and cognitive degenerative processes^9,62^ and the ability to integrate sensory information decreases, leading to a reduction in balance and therefore a higher risk of falling.

The risk of falling in the elderly has a considerable impact on their quality of life, both on social and economic aspects: hospitalisations due to falls count annually around 32.9% of the total.^5^ Moreover, after a fall, subjects report problems with *mobility* (70%), *self-care* (41%), *daily activities* (64%), and *anxiety/depression* (28%), showing how falls lead to functional limitation and a general detrimental impact on the quality of life.^28^

Interest on the body’s ability to maintain equilibrium has grown in recent years, leading to continued advances in the methods and approaches used to quantitatively assess it. From a biomechanical point of view, balance control is assessed by analysing the variation of the Centre of Mass (CoM), its relationship with the Base of Support (BoS), and the alignment of the Centre of Pressure (CoP) with respect to the Centre of Gravity (CoG).^79^ Traditional posturographic examination is performed on force platforms (considered as gold standard). Wearable inertial sensors have been increasingly used to provide similar metrics.^62^ Stereo-photogrammetric 3D motion capture systems are also used to investigate the control of the entire trunk posture and to obtain additional biomechanical measurements.^64,72^ Through these approaches, it is possible to observe and assess how the impairment of systems involved in human upright posture induces an increase in body sway and leads to greater instability.^3,29,51,53,54^

Sensory deficits lead to a reduction in stability, but can be re-balanced by an increase in sensory information, for example *via* additional auditory, visual or vibrotactile stimulations,^14,31,47,73^ as demonstrated by a vast body of literature, can be used as a complement to rehabilitation strategies to improve or partially restore balance control with minimal interference with common daily activities. In addition to the beneficial effects on pathological or neurological conditions such as Parkinson’s^43^ and Alzheimer’s^23^ diseases, stroke^15^ or sensory impairment,^18,40^ some evidence on the positive effect of sensory stimulation has also been reported for healthy subjects.^1,16,39,55^

Despite the vast literature on this topic however, the great variety of stimulation approaches available make it extremely varied and unstructured. A general uncertainty on the right protocol to use exists and is mainly related to the numerous stimuli characteristics (e.g., frequency, intensity, amplitude, association between different stimulation type).^17,46,60^ Moreover, it is not clear whether there is one stimulus that has a greater influence than another, or whether specific stimulation characteristics are eliciting better effects on balance than others. This uncertainty leads to poor or empirical, if non-existent use of additional sensorimotor stimulation in clinical rehabilitation of pathological conditions and moreover in healthy population.^65^ Acoustic and/or vibratory stimulations could be a significant aid for healthy population with increased risk^47^ (e.g., ageing), with minimal interference with common daily activities.

The authors of this work aimed therefore at collecting, organising, and discussing all the literature to date on the effect of the various acoustic and vibratory stimulation techniques, and the combination of both, available on static upright posture in healthy subjects. Furthermore, this work intends to highlight whether there is any key characteristic of those stimulations that may improve the effectiveness of the intervention on postural stability. The authors want to contribute to the development of innovative and comprehensive rehabilitation approaches combining new technologies alongside traditional rehabilitation protocols.

## Methods

### Literature Search

A database search to the latest available date (last search September 2022) was conducted to identify potentially relevant articles in accordance with the Preferred Reporting Items for Systematic reviews and Meta-Analyses (PRISMA) guidelines.^49^ Four electronic databases (PubMed, Web of Science, Cochrane, and Scopus) were searched, using the following keywords and combination of them: *Postural control, Postural stability, Balance, Upright stance, Auditory cue, Acoustic stimulation, Vibratory stimulation, Acoustic Cue, Vibratory Cue, Vibrotactile stimulation, Healthy, Vibration, Postural Response.* The strings used for the PubMed database have been reported here: [*(postural control) OR (postural stability) OR (postural response) OR (balance) AND (auditory cue) OR (acoustic stimulation) OR (acoustic cue) AND (vibratory cue) OR (vibratory stimulation) OR (vibrotactile stimulation) OR (vibration) AND (healthy)*],[(postural control) OR (postural stability) OR (postural response) OR (balance) AND (auditory cue) OR (acoustic stimulation) OR (acoustic cue) AND (healthy)], [(postural control) OR (postural stability) OR (postural response) OR (balance) AND (vibratory cue) OR (vibratory stimulation) OR (vibrotactile stimulation) OR (vibration) AND (healthy)]. A hand search of reference lists of the retrieved papers was also additionally completed.

### Study Selection and Screening Process

Studies analysing static balance control following vibratory or acoustic stimulation in healthy young and elderly adults were included in this review. Exclusion criteria include: (1) studies involving pathological subjects; (2) studies analysing the effect of sensory stimulation on gait; (3) studies evaluating the effects of stimulation on postural control in conjunction with other experimental conditions (e.g., dual task, sleep deprivation *etc*.). Non-English language papers, other reviews and studies published in books or conference proceedings were also excluded.

## Results

**Figure 1:**
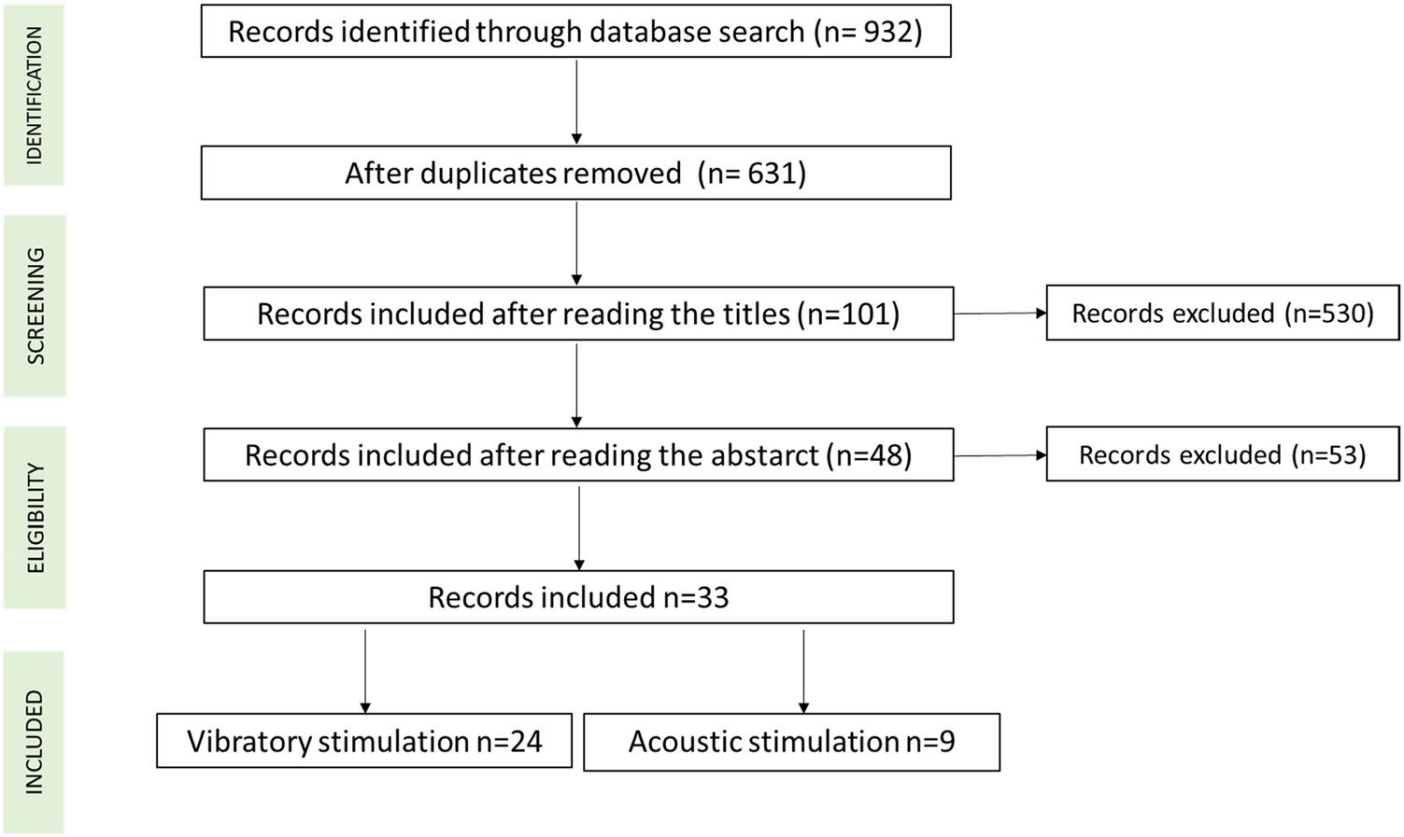
Flow chart of records search and selection process.

After the initial search, 932 articles were found (Fig. 1). The exclusion of duplicates reduced the number of potential articles to 631. From analysis of the titles and abstracts 33 articles were included in this review. To aid the organisation and further presentation of the literature, a subgroup analysis was carried out according to the sensory stimulation approach: retrieved studies include 24 articles utilising vibratory stimulation (see Table 1) and 9 utilising acoustic stimulation (see Table 2). No articles were found which investigated both stimulations.

**Table 1:** Summary of study characteristics for literature concerning vibratory somatosensory stimulation.

Authors and year	Participants		Protocol		Characteristics of the stimulus			Postural assessment	
	Cohort characteristics	Control group	Experimental conditions	Type of sessions	Stimulation device	Stimulation tool positioning	Frequencies and amplitudes	Instrumentation recordings	Outcomes
Ehsani *et al*. 2018	30 participants: 10 mean age 23.30 ± 2.26 years 10 mean age 72.90 ± 2.81 years 10 high risk fall mean age 83.60 ± 9.46 years	No	OE/CE without vibrator OE/CE vibrator—no stimulation OE/CE vibrator + stimulation	2 trials 4 conditions (30 s each)	Focal vibrator	Gastrocnemius	30 Hz 40 Hz 1 ± 0.002 mm amplitude	Force platform Tri-axial gyroscope	Local control slope Central control slope Local control time interval CoG- sway
Ito *et al*. 2014	25 participants: (13 M, 12F) Mean age 46.0 ± 3.0 years	No	Stimulation with CE on: Lumbar multifidus Gastrocnemius	1 trial 6 conditions (30 s each)	2 vibrators	Lumbar multifidus Gastrocnemius	30 Hz 60 Hz 240 Hz	WII balance	CoP AP displacement
Kinnaird *et al*. 2016	8 participants: (3M, 5F) Mean age 65 ± 2 years	No	Attractive and repulsive cues	1 trial 2 sessions (5 min each) in two different days	4 Vibrators (Tactors C2)	Right/left internal oblique Right/left external oblique	80 Hz	Dynamic posturography testing (Equitest)	AP root mean square, Time-In-Zone 95% confidence-interval Ellipse
Lee *et al*. 2012	11 participants: (7M, 4F) Mean age 22.9 ± 4.8 years	No	Stimulation with CE on: Right and left internal obliques Right and left external obliques Right and left erector spinae 50 Hz all locations	2 trials 7 conditions (15 s each)	6 Vibrators Tactors (C2)	Right/left internal oblique Right/left external oblique Right/left erector spinae	250 Hz	Force platform, Inertial measurement unit	Root mean square Postural shift vector Power spectral density
Lee *et al*. 2013	11 participants: (7M, 4F) Mean age 22.9 ± 4.8 years	No	Stimulation with CE on: 250 Hz right/left internal oblique 250 Hz right/left external oblique 250 Hz right/left erector spinae 50 Hz all locations	2 trials × 7 conditions (15 s each) for each tactor type	12 Vibrators	Right/left internal oblique Right/left external oblique Right/left erector spinae	250 Hz	Inertial measurement unit	Root mean square Postural shift vector Power spectral density
Lipsitz *et al*. 2015	12 participants: (1M, 11F) Mean age 73.8 ± 1 years	No	OE/CE	4 trials 2 conditions (60 s each)	Vibratory foot insole	Feet plant	0–70-85% of each participant threshold value	Force platform	CoP velocity CoP distance Sway speed Sway area
Zhou *et al*. 2016	12 participants: (1M, 11F) Mean age 73.8 ± 1 years	No	OE/CE	4 trials 2 conditions (60 s each)	Vibratory foot insole	Feet plant	0–70-85% of each participant threshold value	Force platform	AP/ML multiscale Entropy
Martin *et al*. 2015	10 participants: (5M, 5F) Mean age 22.0 ± 3.1 years	No	Stimulation with CE: Single vibration Co-vibrations Sequential vibrations	3 sessions: 4 trials × 6 single vibration (15 s) 4 trials × 5 co-vibrations (15 s) 4 trials × 2 sequential vibration (5 s)	6 Vibrators (tactors C2)	Right/left internal oblique Right/left external oblique Right/left erector spinae	250 Hz 200 *μ*m	Force platform	CoP displacement
Toosizadeh *et al*. 2018	30 participants: 10 mean age 18–30 10 ≥ 65 healthy 10 ≥ 65 high fall risk	Yes	Stimulation with OE and CE: No vibrations No stimulations 30 Hz 40 Hz	2 trials 4 conditions (30 s each)	Focal vibrator	Gastrocnemius	30 Hz 40 Hz	2 wearable sensors including Tri-axial gyroscope	Sway velocity Body tilt Ankle/hip AP sway COG sway
Lapole *et al*. 2012	17 participants: mean age 27.3 + 3.5 years	Yes	Sitting/standing position	1 h	Vibrators	Achilles tendon	50 Hz	EMG	SOL Hmax/Mmax amplitude ratios
Uimonen *et al*.1995	30 participants: (30 M) Mean age 17–21 years	No	OE/CE on: No vibrations 50 Hz 90 HZ Stance after vibrations	1 trial 8 conditions (30 s, 15 s,10 s,9 s)	2 Vibrators (DC motors)	Gastrocnemius	50 Hz 90 Hz	Force platform	Body sway length Max ML displacement Max A/P displacement
Tjernstrom *et al*. 2001	12 participants: (6M,6F) Mean age 26 years	No	OE/CE	5 days (1 h training) repeated after 90 days	Vibrators	Gastrocnemius	85 Hz	Force platform	Variance body sway Total torque variance Low frequency torque variance High frequency torque variance
Gurfinkel *et al*. 1995	12 participants	No	/	90 s	Vibrators	Tensor fasciae latae	40 Hz; 66 Hz	Potentiometer	CoG displacement
Fransson *et al*. 2004	70 participants: (18M, 12F) mean age 37.8 years (13M, 27F) mean age 76.4 years	No	OE/CE	1 Pseudorandom binary sequence for each condition	Vibrators	Gastrocnemius	85 Hz	Force platform	Torque Feedback control, Postural adaptation Stimulus adaptation
Priplata *et al*. 2003	27 participants: (10M, 5F) mean age 23 years (4M, 8F) mean age 73 years	No	CE with No vibration With vibration	Young: 10 Trials (30 s) × 2 conditions elderly: 5 trials (30 s) × 2 conditions	Vibratory foot insole	Feet plant	90% of each participant threshold value	3D motion system Vicon	Mean radius Swept area Maximum radius AP range ML range Critical mean square range (Δr^2^); Effective long term diffusion coefficient; Long-term scaling exponent
Polonyova *et al*. 2001	15 participants: (7M, 8F) mean age 23.3 years	No	CE; no vibration 40 Hz 60 Hz 80 Hz 100 Hz	16 conditions (20 s) (4 muscles (TA and GA dx and sx) × 4 vibration frequencies	Vibrators (DC motors)	Gastrocnemius (right, left) Tibialis Anterior (right, left)	40 Hz 60 Hz 80 Hz 100 Hz	Force platform	ML and AP COP displacement
Thompson *et al*. 2010	12 participants: (5M, 7F) mean age 26.8 years	No	CE normal surface/tilt support surface toes-up/tilt support surface toes-down No vibration Achille’s tendon vibr (ATV) ATV + Forefoot vibr. (FFT) ATV + Rearfoot vibr. (RFT) FFT RFT	5 vibratory combinations; 3 trials for vibration without RFV and FFV; 5 trials with RFV and FFV	Plantar Vibrators 2 vibrators (custom-made)	Achilles tendon Forefoot Rearfoot	80 Hz; 1.5 mm amplitude	2 Triaxial force plates 3D motion system Vicon	ML and AP COP displacement
Čapičíková *et al*. 2006	17 participants (7M,10F) mean age 26.5 years	No	CE	6 trials: 2 for each vibration duration	Mechanical Vibrators (DC motors)	Soleus	60 Hz; 1 mm amplitude	Force platform	AP COP displacement
Abrahamova *et al*. 2009	18 participants: (6M, 3F) mean age 26 years (7 M, 2F) mean age 63 years	Yes	CE: 40 Hz 60 Hz 80 Hz	9 trials (10 s): 3 for each vibration frequency	Vibrators (DC motors)	Achilles tendon	40 Hz 60 Hz 80 Hz	Force platform 3D motion system BTS	AP COP displacement
Kiers *et al*. 2014	20 participants: mean age 36 years (± 15)	No	CE: firm surface/foam surface; Lumbar Paraspinal vibration Triceps Surae vibration	4 trials for 6 conditions	Vibrators	Lumbar Paraspinal Triceps Surae	70 Hz; 0.5 mm amplitude	Force platform	CoP displacement (dP) COP Velocity (dV)
Mohapatra *et al*. 2012	9 participants: (3M, 6F) mean age 23.9 years (± 0.9)	No	OE/CE; OE + vibration (VEO); CE + vibration (VEC);	5 trials (5 s) for each condition	Vibrators (custom-made)	Achilles tendon	90 Hz; 1 mm amplitude	Force Platform Accelerometer EMG	CoP displacement EMG data
Gomez *et al*., 2009	18 participants: (9M, 9F) mean age 25.1	No	OE- vibration on the neck OE- vibration on the calf CE- vibration on the neck CE- vibration on the calf	200 s of pseudorandom binary sequence for each condition	Vibratory stimulation device (DC motor)	Neck Gastrocnemius	85 Hz; 1 mm amplitude	Force platform	Mean angular position of head, shoulder, hip and knee
Vuillerme *et al*., 2002	10 participants mean age 23.9 years	No	CE	10 trials 5 under fatigue condition 5 no fatigue condition	Mechanical Vibrators	Ankles Gastrocnemius tendons Soleus Tibialis anterior	85 Hz; 1 mm amplitude	Force platform	CoP speed CoP displacement
Naka *et al*., 2015	13 participants (6M-7F) mean age 24.4	No	CE-OE	Two sessions (single vibration/simultaneous vibration) 3 Trials for each frequency for each session	Mechanical vibrators	Forefoot sole Triceps surae	From 1 to 60 Hz; 0.5 mm amplitude	Force platform	CoP AP

*OE* open eyes, *CE* closed eyes, *M* males, *F* females, *CoG* Centre of Gravity, *CoP* Centre of Pressure, *AP* anteroposterior, *ML* mediolateral.

**Table 2:** Summary of study characteristics for literature concerning acoustic stimulation.

Authors and year	Participants		Protocol		Characteristics of the stimulus			Postural assessment	
	Cohort characteristics	Control group	Experimental conditions	Type of Sessions	Stimulation device	Stimulation tool positioning	Frequencies and intensity	Instrumentation recordings	Outcomes
Tanaka *et al*. 2010	12 participants: 7 (21.9 + 1.5 years) 5 (68.9 + 4.0 years)	No	OE/CE Normal surfaces/soft surface Clockwise /counterclockwise stimulation With/without acoustic stimulation	1 trial 8 conditions (20 s each)	Headphones	In ears	White noise 50 Db	Force platform	CG-AREA MAX AP/ML CoP displacements
Seiwerth *et al*. 2018	30 participants: 14F, 16M (29.6 ± 11.2 years)	No	In quite with earplugs With noise	50 steps 3 trials 2 conditions	Loudspeaker	1.85 m away from the subject	White noise 65Db SPL	Cranio-corpography, markers on L/R shoulder and A/P vertex head	Fukuda Test, Distance of displacement, Angle of displacement; Angle of rotation
Anton *et al*.2019	30 participants 18F, 12M, Average age 25 years	No	Reference condition WNIn/WNCn Earplugs	5 trials 4 acoustic conditions (20 s each)	Loudspeaker	2mt in front of the subjects: (1) Room with Short Reverberum (2) Room with Long Reverberum;	WNIn; WNCn; from 80 Hz to 20 kHz	Vertiguard system	Angular velocity of trunk movements
Gandemer *et al*.2017 1 step	30 participants 22M (27.6 ± 4.7 years) 13F (25.8 ± 3.4 years)	No	No sound; 1 sound source; 2 sound sources 3 sound sources Normal/anechoic room	6 trials 4 conditions × 2 room	Loudspeaker	Below the subjects, above the level of the head: (1) Normal room (2) Anechoic room	Ecologic sound sources (average amplitude: 45.5 dBA)	Force platform	Position of CoP Area within the sway path, Mean sway velocity
Gandemer *et al*.2017 2 step	30 participants 15M (28.1 ± 4.5 years) 13F (28.2 ± 4.9 years)	No	No sound 3 isolated ecological sources 10 isolated ecological sources; Immersive environment Normal surface/Foam surface	5 trials 4 conditions × 2 surface	Loudspeaker	3-D positions of different sound sources in a standard room	Background noise (30 Dba) 3 isolated ecological sources (45,5 Dba); 10 isolated ecological sources (50 Dba)	Force platform	Position of CoP, Area within the sway path Mean sway velocity
Zhong *et al*. 2013	19 participants 11F, 8M Average age 27	No	OE/ CE With/without sound	2 trias 5 block (40 s) Fukuda Test: 100 steps 10 repetition	Loudspeaker	Tandem Test: 1 m in front of participants Fukuda Test: 2 m in front of participants	White noise: 75 Db (Tandem Test), 65 Db (Fukuda Test)	Infrared system	Body sway, Angular deviation
Ross *et al*. 2016	30 participants 15 (19.87 ± 2.10 years) 15 (78.67 ± 7.73 years)	No	OE/CE White Noise/silence	5 trials 4 conditions (30 s each)	Headphones	In ears	White noise 75 Db	Force platform	AP/ML SWAY Radial sway
Maheu *et al*. 2016	14 participants 10F, 4M (32.77 ± 15.46 years)	No	OE/CE Firm surface/Foam Surface With/Without noise	4 trials 4 conditions (60 s each)	Double hearing protection Loudspeaker	In a room, 1 m behind the subject	Pink noise (100 Hz, 4 Hz)	Force platform	Sway area Sway velocity
Vitkovic *et al*. 2016	50 participants 10M, 40F Average age 28.84	No	OE/CE Normal Surface/Foam Surface Normal room A sound-treated room with the subject earplugs; A sound-treated room with a continuous white noise A sound-treated room with a moving noise	4 trial 4 conditions	(1) Loudspeaker (2) Earplugs	Front speaker 106 cm away, moving noise in the room	White Noise/between 60 and 70 dB	Force platform	CoP path length
Park *et al*. 2011	11M Average age 22	No	12 conditions (3 for each level sound pressure and 4 for each frequency)	1 trial 12 conditions (20 s each)	Headphones Sound Generator	In ears	1000, 2000, 3000, 4000 Hz/45Db, 90 Db 120 Db	Force platform	CoP position; Length of p AP and ML sway

*OE* open eyes, *CE* closed eyes, *M* males, *F* females, *CoG* Centre of Gravity, *CoP* Centre of Pressure, *AP* anteroposterior, *ML* mediolateral, *WNIn* white noise interrupted, *WNCn* white noise continuous.

For both vibratory and the auditory stimulation, the following information were retrieved and presented:

- *Participants* number and cohort characteristics
- *Protocol* type of session and experimental conditions
- *Characteristics of the stimulus* stimulation device, positioning relative to the participant and environmental condition, stimulation intensity and frequency
- *Postural assessment* device used, assessment parameters

### Literature on Vibratory Stimulation

#### Participants

The sample size in these studies it is generally small, it ranges from 8^4,33^ up to maximum of 70 participants.^20,22,71,73^ The overall age of the subjects recruited in the selected studies is also particularly heterogeneous. Most participants belonged to a middle/young age group (from 18 to 60 years). Two studies^38,81^ investigated the effects of vibratory stimulation on senior participants (90 years old), while in other two studies^20,71^ the cohorts included elderly adults (> 65 years old) with high risk of falling. Some of them compared an elderly population with an young one.^2,20,22,58,71^

#### Experimental Protocols

The current literature can be organised into three main groups, according by the number of carried trials: those with a single trial,^27,30,34,69,73^ two trials^11,20,33,35,37,71^ and four trials^38,44,81^ for each condition. Priplata *et al*. conducted their study using 10 trials for each condition in the young, and only 5 in the elderly.^58^ Our analyses highlighted that trials were generally conducted during the same day with non-substantial differences in duration and number or breaks between each trial; only in two studies the vibratory stimulation was applied for 1 h,^27,34^ while two other studies applied the stimulus according to a pseudorandom binary sequence (PRBS) providing different durations to each stimulation.^7,22,25^ Table 1 reports the details of all the studies.

Open eyes/closed eyes (OE-CE) approach was the most frequently adopted experimental condition to assess differences with and without the visual feedback. Some authors also explored additional experimental conditions, e.g., examining postural differences with and without vibratory stimulation or applying more than one frequency of stimulation to the subject. Furthermore, two studies included experimental conditions to alter proprioception, either through an oscillating surface or a sponge under the feet.^32,68^

#### Characteristics of the Stimulus

In the majority of the studies, the stimulation device is referred as *vibrator*,^2,22,27,30,32,48,70^ mechanical vibrators,^11,50,75^ focal vibrator^20,71^ or generically *stimulator*. In four articles^33,35,36,44^ the authors resorted to the use of particular types of *tactors* (C2-EAI Inc.) and *tactaid*: tactors attached to the subject's skin with medical tape. In four studies^38,50,58,81^ the vibratory stimulus was generated by a vibratory insole located under the subject’s foot plant.

Analysing the differences between the positioning of the stimulation device on to the subjects' bodies, essentially two areas of the body were subjected more to vibratory stimulation: the legs (mainly the gastrocnemius) and the trunk (on the neck, left and right internal/external oblique and erector spinae). Less commonly, other muscles exposed to the vibratory stimulation were the tensor of fasciae latae,^27^ the lumbar multifidus,^30^ the soleus,^11,75^ the tibialis anterior^57,75^ and the tricipes surae.^32,50^ Additionally, several studies applied the vibratory stimulations on the Achilles tendon directly.^2,34,48,68^

The range of frequencies adopted varied from 30 up to 500 Hz. In almost all the studies, the authors performed protocols of stimulation using different frequencies in different sessions.

The amplitude of the mechanical stimulus was not always reported. Of the selected articles, most authors used stimulation 1 mm.^11,25,48,75^ Ito *et al*. reported a vibratory stimulation with amplitude of 1.6 mm,^30^ and Thompson of 1.5 mm,^68^ while Kiers and Naka used an amplitude of 0.5 mm^32,50^; one study used a very small amplitude 200 *µ*m.^44^ In two cases^38,81^ the authors applied vibratory stimulation at fraction of the stimulus perceptibility threshold (i.e. 0, 70, and 85%) for individual subjects but they did not specify any characteristics of the stimulus (e.g., frequency or amplitude).

#### Recording Equipment

Regarding the recording tools adopted in the studies, the force platform is the most widely used tool to assess postural information with different information analysed (e.g., stabilogram diffusion function). Other recording tools included: 3D motion systems^58,68^; an accelerometer^48^; a tri-axial gyroscope^20,71^; a WII balance platform^30^; a set of computerised dynamic posturography tests^33^; a potentiometer^27^; IMU (inertial measurement unit)^35,36^ and an electromyography (EMG).^34^

#### Outcomes

In a fair number of articles, the outcomes investigated were the variation of the CoP, its displacement in the anterior/posterior and medio/lateral direction^2,11,30,48,50,57,68,75^ and its velocity.^32,38,48,75^ Some outcomes investigated the CoG variations, such as CoG sway, the displacement of the projection of CoG,^27^ the sway velocity which analyses the CoG sway distance, divided by the test duration (cm/s) and the body tilt which represents the average of CoG location during the trial.^71^ Some other authors used other quantities, such as the root mean square of the A/P sways,^33,35,36^ the power spectral density of the sway (PSD)^35,36^ or the multiscale entropy (MSE), to quantify the complexity of the postural sway.^81^ Furthermore, Kinnaird^33^ and Lee^36,37^ used the 95% confidence interval ellipse to analyse postural sway by defining their outcomes *postural shift vector*^33^ and *sway area*^35,36^ respectively. Ehsani *et al*.^20^ evaluated parameters such as the local control slope and the central control slope which provided information about the characteristic of the body sway. Lastly, Gomez *et al*., evaluated the antero-posterior body position calculating the mean angular position of the head, shoulder, hip and knee.^25^

### Literature on Auditory Stimulation

#### Participants

Unlike the articles on vibratory stimulation, the number of participants recruited was found to be slightly higher: samples included at least 11 subjects^52^ but no more than 50.^74^ There was a prevalent presence of healthy young group aged between 18 and 38 years old. Two studies analysed elderly people with a mean age of 68.9 + 4.0 years old^67^ and 78.67 years old.^61^ Furthermore, only one paper considered a control group, comparing the differences in postural responses between young and old subjects.^67^ Table 2 reports all the details of the selected papers.

#### Experimental Protocols

Focusing on the experimental execution, the experiments were generally conducted on the same day and the number of trials varied according to the different experimental conditions ranging from 1^52,67^ to 6 trials.^24^ For 3 out of the 9 selected papers we found 5 trials for each condition.^6,24,61^ The duration of each trial did not exceed one minute. In each study, authors decided to adopt more than one experimental condition, which may be categorized into three main groups: (1) open eyes/closed eyes; (2) with and without auditory stimulation; (3) recording on a normal surface and on a foam surface to verify whether a change in proprioception may lead to a further alteration in the response to the auditory stimulus.

#### Characteristics of the Stimulus

The authors decided to pursue two different approaches: those who decided to use loudspeakers, which ensure the sound diffusion throughout the room, and those who used earphones, which isolate the subject from the external environment and produce sound only at the level of the subject's ears. The auditory stimulus is classified mostly according to its intensity, being for most of studies white noise (i.e. wideband). The analysis of the selected studies showed that some authors reported the auditory impulse at a specific decibel (Db) level, thus measuring the noise level in an absolute way.^52,61,67,80^ Others, such as Gandemer^24^ used DbA, which accounts for the distance of the subject from the sound source. In only three papers^6,41,52^ the authors specified specific frequencies for the auditory stimulus.

#### Recording Equipment

Even for auditory stimulation, it emerged that the force plate is by far the most widely adopted recording tool. Other recording instruments used were: Vertiguard system, which is a small box fixed to the subject’s waist trough an elastic band that measures the trunk’s momentary angular velocity in the A/P and M/L directions to the hip^6^; a cranio-corpography positioned on the participant’s head which provides an image of subject’s movement pattern^63^ and infrared-system for the position of the head.^80^

#### Outcomes

The majority of the outcomes assessed the different CoP variations depending on the acoustic stimulation such as the CoP path length,^52,74^ and the length of A/P and M/L sway,^52^ and the area within the sway path which assessed the position of the CoP.^67^ Other authors considered velocity as their principal outcome: Tanaka *et al*. analysed the mean sway velocity, while Anton *et al*.^6^ the angular velocity of trunk movements. Other outcomes included are: the distance of displacement, the angle of displacement and the angle of rotation which were the three main results of the Fukuda test.^63^

## Discussion

The literature is quite varied: each author pursued different paths in terms of parameters assessed, stimulation instruments and their location, resulting in the development of extremely varied scenarios. In the following we have tried to extract clear take-aways from the existing literature for each of the stimulation approaches and the combination of both.

### Effects of Vibratory Stimulation

Out of 24 papers analysed, different results emerged on the effect of vibratory stimulation on balance. Some studies shown that vibratory stimulation can promote a reduction of postural sway,^38,70,71^ others highlighted an increase in sway with forward or backward body tilt.^2,7,11,81^ Reductions in body sway are mainly appreciable in elderly population with high risk of falling, while less than 10% of young and older subjects showed a small reduction of post stimulation sways.^20^ Some authors found the most significant results when the frequency of stimulation applied, regardless of the location, was 30 Hz^20,30,81^ and this may be explained as lower frequencies seem to act directly on somatosensory system^26^ and especially the Meissner corpuscles.^30^ Kinnaird *et al*. highlighted reduced AP oscillation and a smaller 95th percentile confidence interval ellipse obtained using stimuli producing an opposite sway (i.e. when the subjects moved away from the vibratory stimulus^33^). This may be explained from a cognitive point of view, as the stimulation may have been perceived as a threat, so the natural reaction is to move away from it. Lee *et al*.,^37^ found that young adults respond with an increased postural shift (with higher RMS sways during vibration compared to pre and post vibration) in the direction of the stimulus but no change in CoP displacement. Similar results were also found by Martin *et al*.,^44^ who reported that vibration induced a trunk inclination, but neither the 95th percentile confidence interval ellipse of the CoP and the CoP shift vector changed significantly during vibration compared to the pre vibration period or between two consecutive stimulations. These results may seem in contrast one another, although an increase in CoP displacement may not necessarily be detrimental to stability, especially if this is accompanied by an increase in muscular activity. Increased muscular activation may allow for a stronger movement response (thus COP and COG displacement), while also contributing to increase stability with counteracting involvement during postural perturbations.

#### Stimulation Targets and Postural Response

Literature analysis also revealed a variety of target locations of vibratory stimulation, which influence the postural control response.^25^ All the article analysing the effect of vibration on the Achilles tendon highlighted a backward tilt of the body. According to Abrahamova *et al*., body tilt seems to depend on stimulation frequency and age.^2^ They showed that older participants respond to Achille’s tendon vibration with a greater inclination compared to young ones, and that this inclination increases with the increase of frequency of stimulation. This result is intuitively confirmed by the different trunk posture in the two groups. In fact, although the participants had a similar biomechanical response at leg level, the trunk position in elderly followed the direction of the tilt of the legs, while displaying a compensatory movement with an increased hip flexion in young participants. This compensation allowed the young group greater verticality during the stand position and a better postural adaptation.

Vibration of the distal tendon of tibialis anterior and extensor digitorum longus causes an altered proprioception (*illusionary sensation*) of the lower-extremities; this influences the nature of the information coming from the neuromuscular spindles to maintain balance^59^ and may be used as balance challenge.^37^ However, this seems to be age dependent: in the elderly population, as the spindle activity is weaker, vibration elicits less *illusory disturbances*, and therefore appear to act on a more tactile proprioceptive level. This might be also the reason why the older population respond better to this type of vibration in posture balance recovery approaches. Noteworthy, according to Ito *et al*., the older population rely much more on proprioceptive information derived from Meissner and Pacini corpuscles to regulate their postural responses with respect to the neuromuscular spindles. Therefore, a training program based on the reinforcement of the proprioceptive skills in the elderly may be helpful in fostering better postural control.

Stimulation at the level of the erector spinae and the internal oblique induce postural shifts oriented in the direction of the stimulus.^35,36^ Martin *et al*.^44^ proved that vibratory stimulation applied on the trunk also seem to elicit or enhance proprioceptive inputs. Cutaneous receptors act as a reference system of the upper body in the space.^44^

Feet plant stimulation has also been reported to reduce of postural sway as well as CoP displacement in several studies.^13,58,77^ In a particular case, vibratory insole stimulation has been found to promote a reduction in ML postural sway^38,81^ (found particularly sensitive to changes in skin somatosensitive sensibility) especially when the stimulation frequency was at 70 or 85% of each subject's threshold value.^38,81^ In this case a stochastic resonance (SR) stimulation was used (a particular low level of white noise to enhance the detection of a weak signal).^13^ In particular the SR emphasises the detection of sub-threshold signals maintaining the responsiveness of biological systems, such as the vestibular, the visual and the somatosensory systems, to external stimulations.^78^ Partial simulation of the foot’s sole produce a body reaction in the opposite direction to that of the stimulation (i.e. a rearfoot vibration produce a forward whole-body tilt with increased flexion in trunk, hip, and ankle).^11^ The nervous system may perceive the vibration as an increase in pressure, hence, responding with a body tilt in the opposite direction to rebalance it to maintain verticality.^68^ Postural adaptations seem faster when stimulations are applied at the neck compared to the calf (most notably with closed eyes).

#### The Long-Term Memory Effect of the Vibratory Stimulation

Tjernstorm *et al*. also evaluated the effect of repeated vibratory stimulation over time. In their study the vibration was applied for 5 consecutive days toward the calf muscle of both legs at a frequency of 85 Hz, and on each successive trial the subject performed better (reduction of the total and low frequency body sway) than the previous^69^ showing an habituation effect. In addition, their effect largely remained at 90 days. These results suggested that vibratory stimulation could promote the development of a long-term memory for postural adjustments.^8,10^

#### Auditory Stimulation

From the 9 papers related to the auditory stimulation included in this review, the main effect that has emerged is that an auditory stimulus plays an important role in postural control with a reduction of the sway oscillations.^24^ This could be considered for therapeutic purposes especially in elderly people at high risk of falling, considering the typical age-related changes in balance.

#### Source Information

Nevertheless, Anton *et al*. suggested that the effects of acoustic stimulation depend on a number of variables such as the structure of the auditory signal, the sensorimotor conditions of the subject and the nature of the surrounding environment (the greater the auditory environment the better the balance).^6^ Auditory stimuli, which provide information about the surrounding environment, can be used as an additional source of information. In fact, some of the studies analysed showed an increase in postural sway when auditory input was reduced or excluded through the use of headphones or soundproof rooms.^24,41,63,74^ According to Maheu and colleagues, participants using headphones to neutralise pink noise emitted by loudspeakers are inclined to engage in sensory reweighting, shifting more attention to visual inputs.^41^

#### Integration of Auditory and Other Sensory System

Several papers have investigated the influence of each of the different sensory systems during the use of an auditory stimulus. All the papers analysing the influence of vision on posture, through the condition of open and closed eyes (OE and CE), agreed that with closed eyes the sways were greater, both in the presence and in the absence of an acoustic stimulus.^61,74,80^ Ross^61^ and Zhong^80^ showed that although there is an increase in stability in the acoustic stimulus condition, both in the basic static condition^61^ and during the Tandem Romberg test and the Fukuda test,^80^ the impact on balance is less than in the visual system. Ross *et al*. also showed that the acoustic stimulus, with both OE and CE condition, had beneficial effects in both the young and elderly population.^61^ With regard to alterations of the somatosensory system, several studies investigated the condition of the foam under the feet in order to reduce proprioceptive information.^6,24,41,67,74^ A reduction in proprioception caused by the sponge, which in turn implies a further reduction in sensory information, led to an increase in postural oscillations. However, in the case of a concomitant auditory stimulus this instability decreases.^74^ Tanaka *et al*. showed a reduction in lateral oscillations in an elderly population subjected to a reduction in the sensory tactile and an acoustic stimulus.^67^ With regard to sensory interference on stability, a further study that is in agreement with the previous works is Kanegaonkar *et al*., according to which postural control is reduced following acoustic stimuli, even in the case of a reduction in other sensory inputs, suggesting that they can be a useful tool for improving the condition of global balance.^31^

#### Stimulus Characteristics

Gandemer *et al*. focused mainly on the type of acoustic stimulus emitted and the number of acoustic sources. In their work, divided into two experiments, using environmental stimuli that are often present in everyday life (e.g. the noise of a car motor, or the sounds of insects) they found that the greater the number of acoustic sources, the greater the stability.^24^ This is in line with the work of Easton *et al*., who confirmed that the more spatial information, the greater the ability to control posture.^18^ Furthermore, Gandemer *et al*., although they analysed a stationary acoustic stimulus, assumed that moving the head during acoustic delivery, recreating a moving stimulus, would result in more spatial information, increasing postural benefits.^24^ This assumption was studied by Vitkovic^74^ and Tanaka^67^ as well. Vitkovic *et al*. showed that among the four conditions tested (with headphones, environmental, stationary sound and moving sound), first of all the conditions of acoustic stimulation were those in which there was greater stability, and that the moving stimulus seems to have a more beneficial effect than the stationary one, even in conditions of sensory deprivation (eyes closed and on a foam).^74^ Tanaka *et al*. also conducted their experiment with a moving stimulus, clockwise and counterclockwise.^67^ As mentioned above, the beneficial effect of the stimulus is present in the elderly population by reducing lateral oscillations, which are the ones most likely to be associated with a high risk of falling.^42^ Although a beneficial effect of such a stimulus is clear, this study did not compare the moving stimulus with a stationary one, which might be able to support the thesis of Vitkovic *et al*.^74^ and Gandemer *et al*.^24^

Another characteristic of the acoustic stimulus investigated was the effect of a continuous or interrupted stimulus. Many of the reported articles showed that continuous noise (white or pink noise)^41,63,74^ is able to increase stability. In disagreement with this is the work of Anton *et al*. who reported an improvement in stability in the case of an interrupted stimulus, and a worsening of the continuous stimulus.^6^ However, this is probably due to the type of analysis performed. In fact, while most of the works carry out a posturographic examination by means of a force platform, they investigated angular velocity through an instrument that is placed on the torso, much closer to the centre of gravity of the body, and therefore probably less sensitive to body oscillations.

#### Stimulation Frequency

Finally, the work of Park *et al*. investigated different frequencies and stimulus pressure.^52^ Comparing four different types of frequencies (1000, 2000, 3000 and 4000 Hz) and three different sound intensities (45, 90 and 120 Db), antero-posterior oscillations increased with increasing frequency, although a stimulus with a frequency of 2000 Hz induced greater stability than all the others, including the lower one (of 1000 Hz). In contrast, sound pressure did not seem to interfere with postural control.

## Conclusions

The literature found is extremely inhomogeneous, and further studies should refer to any review on these topics before designing similar trials. Overall, both stimulations analysed were found to have a positive effect on balance so more research is needed to align those alternative approaches to the traditional ones.

Regarding the vibratory stimulus, the main positive effects were found with a stimulation of the cutaneous receptors, rather than the deeper proprioceptive stimulation which appear to be destabilising. Indeed, better results were obtained with low frequency (30 Hz), which may promote earlier activation of skin receptors, in particular the Meissner corpuscles, than neuromuscular spindles, which are activated more slowly. Moreover, this can be a significant factor in the elderly population, as the neuromuscular spindles can often be compromised with the ageing process. Another advantageous approach could be the use of shoe insoles that exploit the principle of stochastic resonance, which could be beneficial without affecting daily activities. In contrast, other papers highlighted an increase of postural inclinations and sways during a vibratory stimulation. Although these results are usually considered detrimental to stability, it should be considered that, certain types of vibration cause the muscles contraction, which, on the one hand leads to increased movement during standing, but on the other hand leads to an increased ability to compensate and maintain balance during disturbances.

Acoustic stimulation was not found to have the same impact as a somatosensory or visual input, although was also found to improve postural control. Providing information about the environment, we found that the more acoustic information, and therefore the more acoustic sources present, the greater the effect of such a stimulation. Lower frequencies (e.g., 1000 Hz) were also shown to have greater efficacy. Therefore, although it is a useful tool for improving balance, it may be most helpful in case of people with sensory deficits, and therefore with reduced sensory information.
